# Origami-based Building Blocks for Modular Construction of Foldable Structures

**DOI:** 10.1038/s41598-017-13654-z

**Published:** 2017-11-01

**Authors:** Davood Mousanezhad, Soroush Kamrava, Ashkan Vaziri

**Affiliations:** Department of Mechanical and Industrial Engineering, Northeastern University, Boston, MA 02115 USA

## Abstract

Origami, widely known as the ancient Japanese art of paper folding, has recently inspired a new paradigm of design for mechanical metamaterials and deployable structural systems. However, lack of rationalized design guidelines and scalable manufacturing methods has hindered their applications. To address this limitation, we present analytical methods for designing origami-based closed-loop units with inherent foldability, and for predicting their folding response (e.g., folding force, bistability, and area and volume change by folding). These units can be employed as building blocks for application-driven design and modular construction of foldable structures with desired performance and manufacturing scalability.

## Introduction

Origami-based systems^1–3^ can exhibit unique properties such as tunable stiffness^4^, tunable chirality^5^, tunable thermal expansion^6^, programmable collapse^7^, programmable curvature^8^, auxeticity (i.e., having negative Poisson’s ratio)^9–11^, multi-stability^4,10,12^, and self-foldability^13^, making them promising candidates for applications such as reconfigurable architected materials^14^, deployable solar panels^15^, fold-core sandwich panels^16,17^, three-dimensional (3D) cell-laden microstructures^18^, flexible medical stents^19^, flexible electronics^20^, MEMS and NEMS^21^, potentiometric bio-sensing^22^, soft pneumatic actuators^23^, and self-folding robots and structures^24^. While these recent advancements have highlighted the potential of origami-based design approaches^9,10,25,26^, lack of robust and application-driven design guidelines and scalable manufacturing methods has limited their applications. Here, we present an analytical method to create a wide range of origami-based closed-loop units – in the form of polygons – which can be used as building blocks for rational design and modular construction of foldable structures.

**Figure 1:**
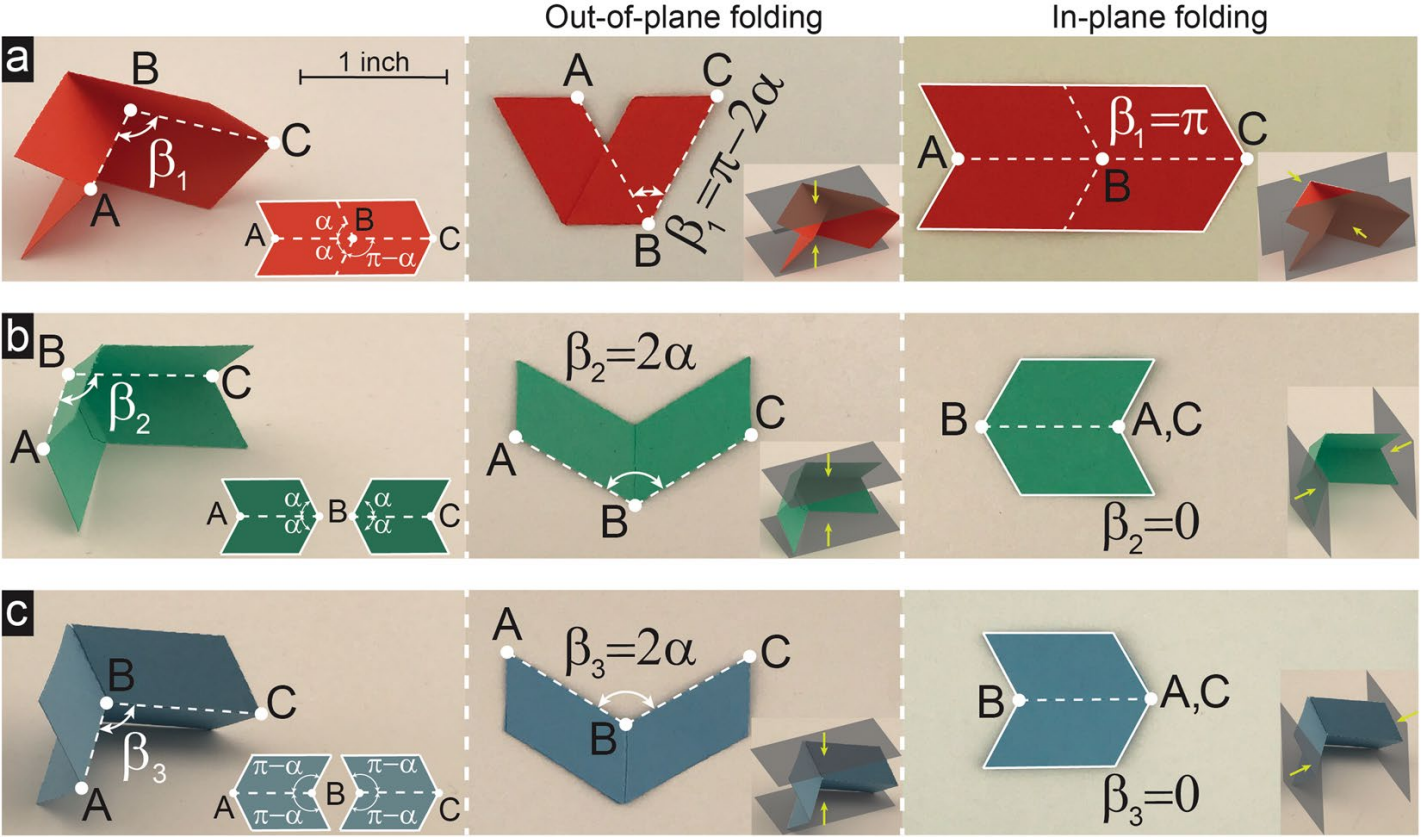
Origami-based fold patterns used to construct closed-loop units. (**a**) The well-known ‘Miura-ori’ fold pattern is constructed out of a single sheet of paper when two identical acute angles, $\alpha$, meet their supplementary angles, $\pi -\alpha$, where folding along four crease lines will result in one mountain and three valley folds (for the sample shown, $\alpha =\pi /3$). Miura-ori is a single DoF foldable origami at which folding is quantified in terms of the angle between the mountain (i.e., line AB) and front valley (i.e., line BC) fold lines (i.e., initially collinear fold lines), ${\beta }_{1}\in [\pi -2\alpha ,\,\pi ]$. Complete folding is achieved at ${\beta }_{1}=\pi -2\alpha$ and ${\beta }_{1}=\pi$ for out-of-plane and in-plane folding directions, respectively. (**b**) and (**c**) The fold patterns shown are constructed when four identical angles meet (acute angles, $\alpha$, or obtuse angles, $\pi -\alpha$), which makes it impossible for them to be folded out of a single sheet of paper. The patterns have one DoF foldability, where folding behavior is characterized by the angle between middle crease lines (i.e., lines AB and BC), ${\beta }_{2}$ and ${\beta }_{3}$. We can show that *β*_2_ and ${\beta }_{3}$ are related to ${\beta }_{1}$ through the relations, ${\beta }_{2}={\beta }_{3}=\pi -{\beta }_{1}$ (see Supplementary Information for details). Complete folding is achieved at ${\beta }_{2}={\beta }_{3}=2\alpha$ and ${\beta }_{2}={\beta }_{3}=0$ for out-of-plane and in-plane folding directions, respectively.

Figure 1a shows a well-known one degree-of-freedom (DoF) origami called Miura-ori, constructed by folding a flat sheet of paper. Miura-ori is characterized by four crease lines which are formed when two identical acute angles, $\alpha$, meet their supplementary angles, $\pi -\alpha$. Then, folding along the crease lines will result in one mountain and three valley folds, which can be quantified in terms of the angle between the mountain (i.e., line AB) and front valley (i.e., line BC) fold lines, ${\beta }_{1}\in [\pi -2\alpha ,\,\pi ]$. ${\beta }_{1}=\pi -2\alpha$ and ${\beta }_{1}=\pi$ represent the extreme cases at which the origami is at fully-folded configurations under out-of-plane and in-plane folding directions, respectively, as shown in Fig. 1a. To enable creating a wide range of foldable designs, we now define two origami-based crease patterns which (in contrast to Miura-ori) cannot be made out of a single sheet of paper (see Fig. 1b,c). This means that an adhesive material (e.g., glue) is needed for attaching the parts together (see Supplementary Information for details). Crease pattern shown in Fig. 1b is constructed by connecting together four identical acute angles, $\alpha$, resulting in a one DoF pattern at which the fold pattern can be characterized by four valley folds with ${\beta }_{2}\in [0,\,2\alpha ]$ as the angle between front (i.e., line BC) and rear (i.e., line AB) valley fold lines. Similarly, the crease pattern shown in Fig. 1c forms when four identical obtuse angles, $\pi -\alpha$, meet. This also results in a single DoF pattern at which the fold pattern is characterized by two mountain and two valley folds with ${\beta }_{3}\in [0,\,2\alpha ]$ as the angle between the two mountain fold lines (i.e., lines AB and BC). We should note that ${\beta }_{2}$ and ${\beta }_{3}$ are two variables that control the folding of these two crease patterns, with ${\beta }_{2}={\beta }_{3}=2\alpha$, and ${\beta }_{2}={\beta }_{3}=0$, represent the extreme cases at which the patterns are at fully-folded configurations under out-of-plane and in-plane folding directions, respectively (see Fig. 1b,c). Interestingly, we can show that ${\beta }_{2}$ and ${\beta }_{3}$ are related to ${\beta }_{1}$ through the relations, ${\beta }_{2}={\beta }_{3}=\pi -{\beta }_{1}$ (see Supplementary Information for details).

Next, we propose an analytical method for designing foldable closed-loop units – in the form of polygons – with one DoF by attaching at least two different crease patterns presented in Fig. 1. For the three crease patterns introduced in Fig. 1, the angle between the middle crease lines (shown by dashed lines) can be presented as $\beta$, and $\pi -\beta$ (we use $\beta$ instead of ${\beta }_{1}$ for the sake of simplicity). These angles, along with their explementary angles, $2\pi -\beta$ and $\pi +\beta$, serve as internal and/or external angles of the final configurations of closed-loop units. For each polygon with $n$ sides, the summation of internal angles must be equal to $(n-2)\,\pi$, where $n$ is the number of fold patterns used to construct the polygon. Denoting ${m}_{1}$, ${m}_{2}$, ${m}_{3}$, and ${m}_{4}$, as the number of internal angles, $\beta$, $2\pi -\beta$, $\pi -\beta$, and $\pi +\beta$ (i.e., ${m}_{1}+{m}_{2}+{m}_{3}+{m}_{4}=n$), we can now present the following geometrical relation:

1 $${m}_{1}\beta +{m}_{2}(2\pi -\beta )+{m}_{3}(\pi -\beta )+{m}_{4}(\pi +\beta )=({m}_{1}+{m}_{2}+{m}_{3}+{m}_{4}-2)\pi .$$

Since the right-hand-side of this equation is a constant for an arbitrary closed-loop unit, to achieve a foldable configuration, the left-hand-side must be independent of the folding variable, $\beta$. This yields, ${m}_{1}-{m}_{2}=2$, and ${m}_{3}-{m}_{4}=2$, meaning that $n$ $(={m}_{1}+{m}_{2}+{m}_{3}+{m}_{4})$ must be an ‘even integer’ greater than or equal to four. Furthermore, we can show that Equation (1) results in $(n/2)-1$ solutions for $({m}_{1},\,{m}_{2},\,{m}_{3},\,{m}_{4})$ for an $n$-sided closed-loop unit (see Supplementary Information for details). Based on this, $n=4$ results in the smallest configuration in the shape of a ‘quadrangle’ with two internal angles of $\beta$ and the other two internal angles of $\pi -\beta$ [i.e., $({m}_{1},{m}_{2},{m}_{3},{m}_{4})=(2,0,2,0)$].

However, foldability is not necessarily guaranteed for a closed-loop unit constructed by an arbitrary combination of the angles that satisfies Equation (1). This means that the ‘sequence’ of these angles in forming the final closed-loop configuration is a key factor that dictates the foldability versus rigidity of the unit. For example, for the quadrangular unit discussed above [which satisfies Equation (1)], the only foldable unit is obtained when identical angles are not adjacent to each other (i.e., the sequence: $\pi -\beta$, $\beta$, $\pi -\beta$, $\beta$), while the other possible configuration (i.e., the sequence: $\pi -\beta$, $\pi -\beta$, $\beta$, $\beta$) will result in a rigid unit. This will be discussed next by deriving a set of mathematical expressions which represent topological constraints on the sequence of internal angles to guarantee foldability.

**Figure 2:**
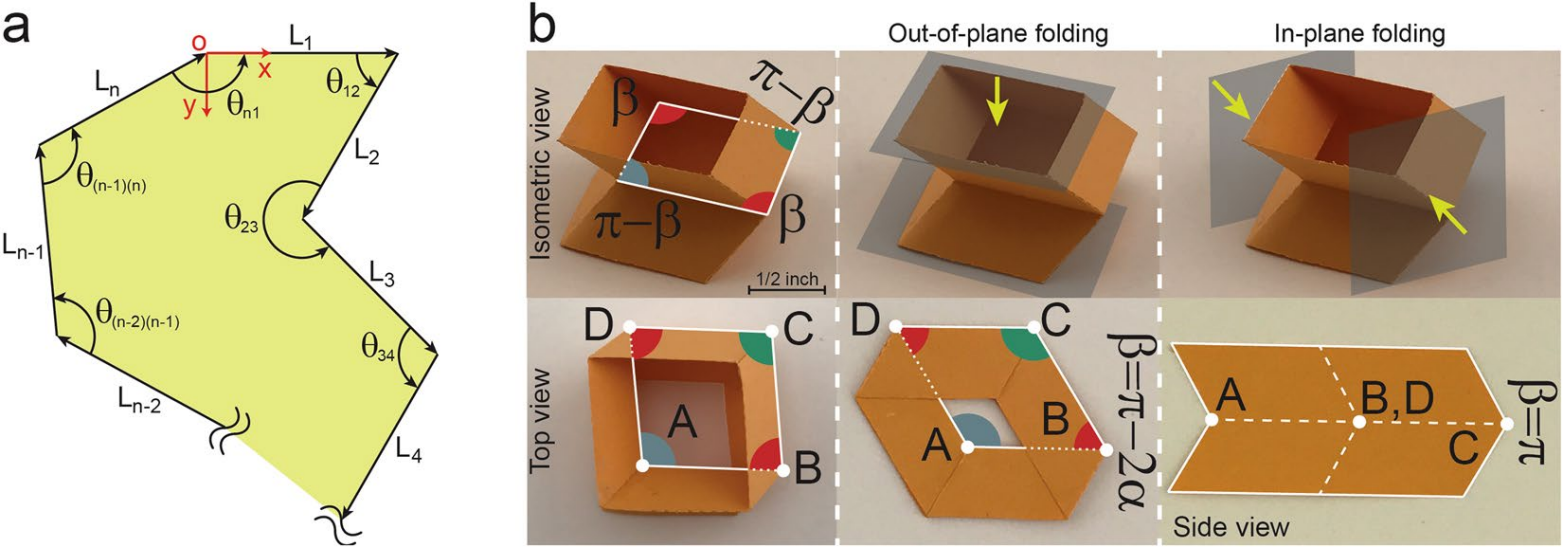
Origami-based closed-loop units. (**a**) Schematic diagram of the middle crease lines of an arbitrary closed-loop unit (constructed by using fold patterns introduced in Fig. 1) with $n$ sides, where ${L}_{i}$ ($1\le i\le n$ is an integer) is the length of the ${i}^{th}$ crease line, ${\theta }_{(j)(j+1)}$ ($1\le j\le n-1$ is an integer) is the internal angle between the ${j}^{th}$ and ${(j+1)}^{th}$ crease lines (positive when counterclockwise), and ${\theta }_{n1}$ is the internal angle between the last and first crease lines (positive when counterclockwise). (**b**) The smallest closed-loop unit with one DoF foldability in out-of-plane and in-plane directions, constructed by using fold patterns introduced in Fig. 1, where $n=4$, ${L}_{1}={L}_{2}={L}_{3}={L}_{4}$ (=1 inch for the sample shown), and the sequence of internal angles ($\pi -\beta$, $\beta$, $\pi -\beta$, $\beta$) [note $\beta ={\beta }_{1}$]. For the sample shown, $\alpha =\pi /3$. Top row shows isometric views of the unit at an intermediate folding level, while the bottom row shows top and side views of the unit at an unfolded as well as fully-folded configurations under out-of-plane ($\beta =\pi -2\alpha$) and in-plane ($\beta =\pi$) directions. We used alphabets, A–D, to label the vertices of the middle crease lines.

Figure 2a shows a schematic diagram of the middle crease lines of an arbitrary closed-loop unit with $n$ sides, where ${L}_{i}$ (with $i$ as an integer varying between 1 and $n$) is the length of the (*i*)^th^ crease line, ${\theta }_{(j)(j+1)}$ (with $j$ as an integer varying between 1 and $n-1$) is the internal angle between the $(j)\,$ ^th^ and (*j* + 1)^th^ crease lines (positive when counterclockwise), and ${\theta }_{n1}$ is the internal angle between the last and the first crease lines (positive when counterclockwise). For the unit to be closed-loop at an arbitrary folding level (i.e., for any value of the angle, $\beta$), the vector summation of the middle crease lines must be equal to zero, i.e., ${\sum }_{i=1}^{i=n}\vec{{L}_{i}}=0$, where $\vec{{L}_{i}}$ is the vector representation of the (*i*)^th^ crease line with magnitude ${L}_{i}$ directing along the corresponding crease line. Considering the coordinate system shown in Fig. 2a, this vector equation can be presented as (see Supplementary Information for details):

2 $$\begin{array}{c}\sum _{i=1}^{i=n}{(-1)}^{i+1}\,{L}_{i}\,cos(\sum _{j=1}^{j=i-1}{\theta }_{(j)(j+1)})=0,\\ \sum _{i=2}^{i=n}{(-1)}^{i}\,{L}_{i}\,sin(\sum _{j=1}^{j=i-1}{\theta }_{(j)(j+1)})=0.\end{array}$$

Using Equation (2), we can now check the validity of all combinations of internal angles given by Equation (1) to see if they result in a foldable closed-loop unit. For example, for a quadrangular configuration with angle sequence ($\pi -\beta$, $\beta$, $\pi -\beta$, $\beta$), applying Equation (2) results in ${L}_{1}=\,{L}_{3}$ and ${L}_{2}=\,{L}_{4}$ to satisfy foldability. However, Equation (2) cannot be satisfied for a quadrangular configuration with angle sequence ($\pi -\beta$, $\pi -\beta$, $\beta$, $\beta$) [see Supplementary Information for details]. Figure 2b shows a foldable quadrangular unit with ${L}_{1}=\,{L}_{2}={L}_{3}=\,{L}_{4}$ = 1 inch. As we mentioned earlier, the closed-loop units presented here cannot be made by folding a single sheet of paper. This means that we must fold multiple sheets of paper (at least two), then attach them together (using an adhesive) to construct the units. Note that the quadrangular unit shown in Fig. 2b retains the single DoF property and foldability of underlying crease patterns in both out-of-plane and in-plane directions (see Fig. 2b – middle and right columns).

**Figure 3:**
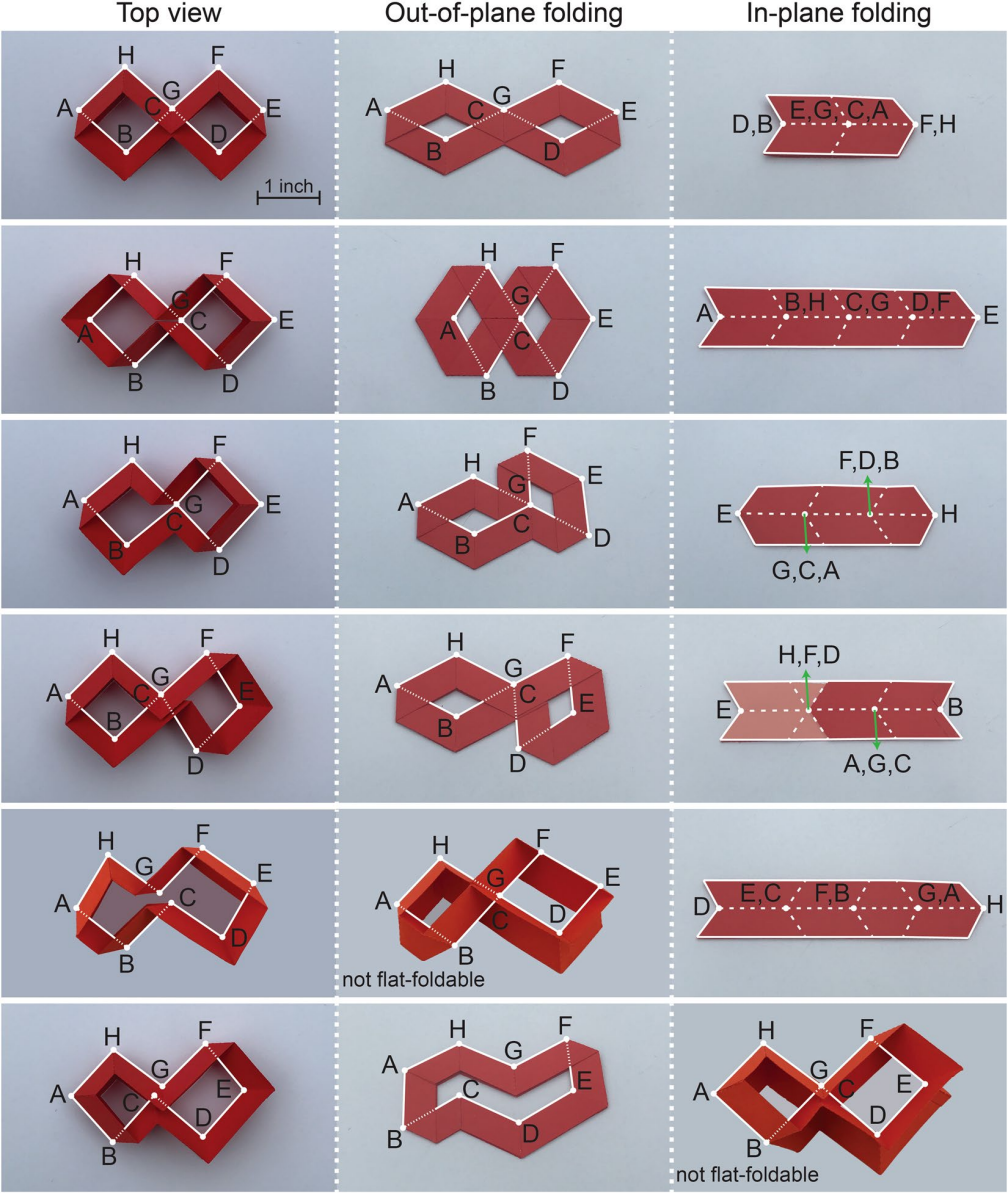
All foldable closed-loop units with $n=8$, constructed by using fold patterns introduced in Fig. 1. All the units are one DoF foldable in out-of-plane and in-plane directions, with ${L}_{1}={L}_{2}=\ldots ={L}_{8}$ (=1 inch for the samples shown). Complete folding in one direction is restricted for the units shown in the last two rows due to geometrical interference (i.e., self-contact). The sequence of internal angles for each unit is given in Supplementary Information. For the samples shown, $\alpha =\pi /3$. Left column shows a top view of each unit at an unfolded configuration, while middle and right columns show top and side views of units at fully-folded configurations under out-of-plane $(\beta =\pi -2\alpha )$ and in-plane $(\beta =\pi )$ directions. We used alphabets, A-H, to label the vertices of the middle crease lines.

Based on mathematical expressions presented here, we developed a MATLAB (MathWorks, Natick, MA) code to first find the solutions of Equation (1) for *n*-sided polygons, then use Equation (2) to select foldable closed-loop unit designs assuming ${L}_{1}={L}_{2}=\ldots ={L}_{n}$. Our results show that $n=6$ results in no possible foldable design, while $n=8$ results in 6 solutions which are shown in Fig. 3 (see Supplementary Information for details on these designs). In this figure, the center of each underlying crease pattern (i.e., vertices of the polygon made by middle crease lines) is labeled by capital letters. The first two configurations can be recreated using tessellations of the foldable quadrangular construction shown in Fig. 2b and thus, do not represent new configurations. Note that all these closed-loop units are one DoF flat-foldable (i.e., capable of transforming into a flat configuration) in out-of-plane and in-plane directions, except the last two configurations which are flat-foldable only in one direction while their foldability in the other direction is restricted due to ‘geometrical interference’ (i.e., self-contact) at points C and G.

Performing the analysis for $n=10$ returns no possible solution, while $n=12$ will result in 141 closed-loop constructions that satisfy both Equations (1) and (2). Here, we present a selected set of these closed-loop units in Supplementary Fig. S2 (see Supplementary Information for details on these designs). Again, all these units are one DoF flat-foldable in both out-of-plane and in-plane directions.

**Figure 4:**
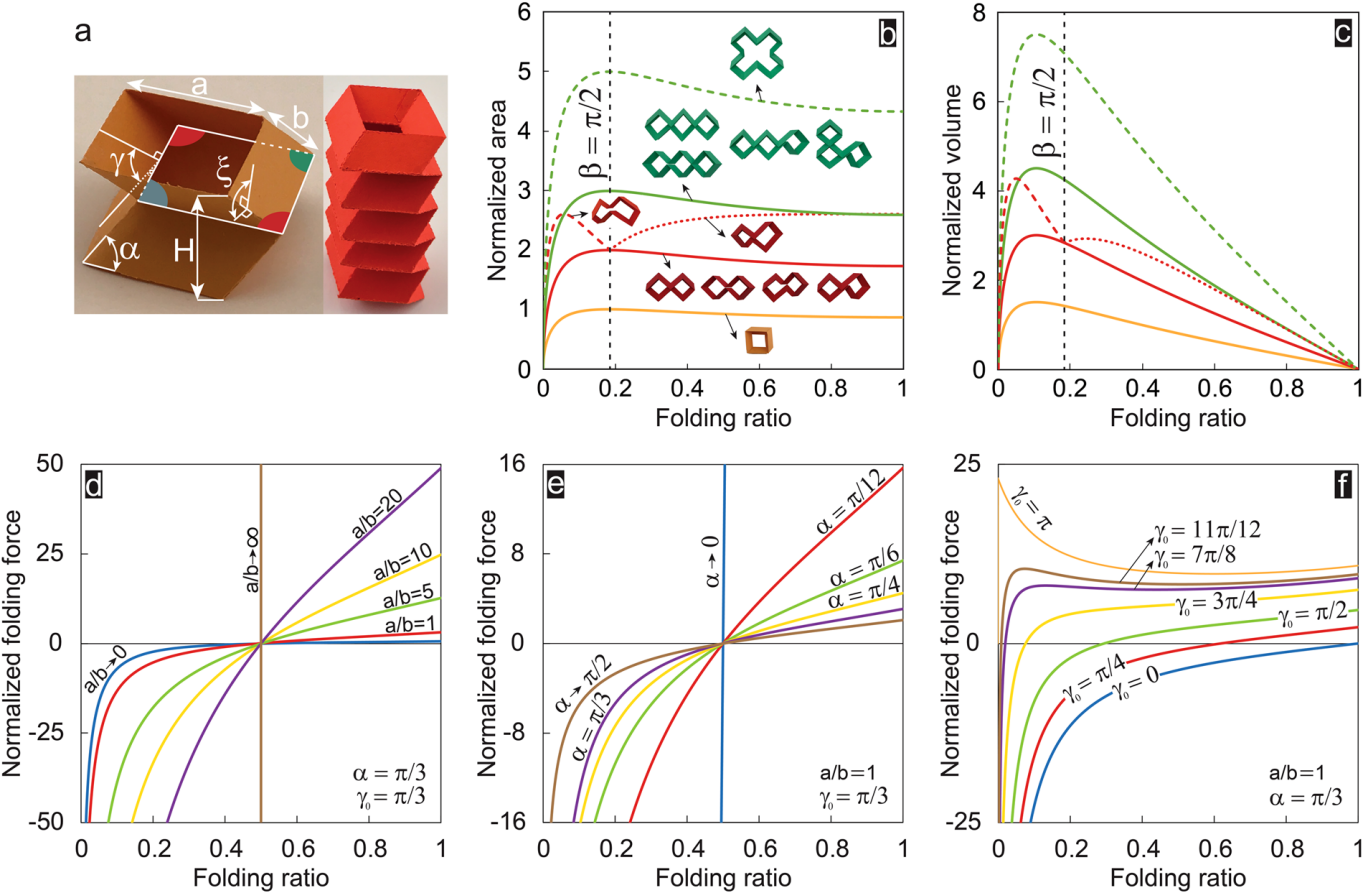
Folding kinematics and kinetics of foldable tubular constructions. (**a**) A sample RVE and the corresponding tubular construction – composed of five RVEs stacked on top of each other in the out-of-plane direction at an arbitrary folding level, with *a* and *b* as side lengths, *H* as the RVE’s height, and dihedral angles, $\gamma \in [0,\,\pi ]$ and $\xi \in [0,\,\pi ]$, as another forms of representation of the single DoF of the unit (similar to *β*). (**b**) and (**c**) Normalized area (defined as the in-plane cross-sectional area of closed-loop units, normalized with respect to ${a}^{2}$) and normalized volume (defined as the volume bounded by closed-loop units, normalized with respect to ${a}^{3}$) versus the folding ratio for all foldable units with $n=4$ and $n=8$, and for a selected set of foldable closed-loop units with $n=12$. (**d**), (**e**), and (**f**) Normalized folding force (defined as the out-of-plane folding force normalized with respect to the spring constant per unit crease length, $k$, and number of polygon sides, $n$, i.e., $F/(k\,n)$) versus the folding ratio for all the RVEs studied in this paper.

The closed-loop units can be stacked up – in out-of-plane direction – to create foldable tubular constructions. Here, we assume an infinite repetition of a ‘representative volume element’ (RVE; same as closed-loop unit), and analytically investigate the kinematics and folding kinetics of tubular constructions by studying the in-plane cross-sectional area, volume, and out-of-plane folding force needed to keep them at an arbitrary folding level. Figure 4a shows an example of an RVE and the corresponding tubular construction – composed of five RVEs stacked on top of each other in the out-of-plane direction – at an arbitrary folding level, where *a* and *b* are side lengths, $H=2b\,sin(\alpha )sin(\frac{\gamma }{2})$ is the RVE’s height, and dihedral angles, $\gamma \in [0,\,\pi ]$ and $\xi \in [0,\,\pi ]$ are another forms of representation of the single DoF of the unit (similar to *β*) which can be derived from the following equations (see Supplementary Information for derivations):

3 $$\begin{array}{c}\beta =\pi -2{cos}^{-1}(\frac{cos\,\alpha }{\sqrt{1-{sin}^{2}(\frac{\gamma }{2}){sin}^{2}(\alpha )}}),\\ \xi ={cos}^{-1}(\frac{1-(1+{cos}^{2}\alpha ){sin}^{2}(\frac{\gamma }{2})}{1-{sin}^{2}(\frac{\gamma }{2}){sin}^{2}(\alpha )}).\end{array}$$

Equation (3) holds true for all closed-loop unit constructions introduced in this paper. Now we quantify folding level by defining a non-dimensional parameter called ‘folding ratio’ as, $1-sin(\frac{\gamma }{2})$, which varies from 0 (i.e., $\gamma =\pi$) to 1 (i.e., $\gamma =0$) [see Supplementary Information for derivation]. Based on this definition, complete folding in in-plane direction corresponds to a folding ratio of 0, while complete out-of-plane folding results in a folding ratio of 1. In-plane cross-sectional area, defined as the in-plane area bounded by closed-loop unit (or tubular construction) [such as the area of the polygon formed by middle crease lines] is equal to ${a}^{2}\,sin(\beta )$ for the unit presented in Fig. 4a and is constant through the height at any folding ratio. The in-plane cross-sectional area for the first four units with $n=8$ and $n=12$, shown in Fig. 3 and Supplementary Fig. S2, can be obtained using similar equations as $2{a}^{2}\,sin(\beta )$ and $3{a}^{2}\,sin(\beta )$, respectively. Moreover, the in-plane cross-sectional area for the last two units shown in Fig. 3 is given as $2{a}^{2}\,sin(\beta )\,[1-cos(\beta )]$ and $2{a}^{2}\,sin(\beta )\,[1+cos(\beta )]$. Also, the last configuration shown in Supplementary Fig. S2 has an in-plane cross-sectional area of $5{a}^{2}\,sin(\beta )$. Figure 4b depicts the variation of ‘normalized area’ (i.e., in-plane cross-sectional area normalized by ${a}^{2}$) as a function of folding ratio for all closed-loop units presented in this paper with $\alpha =\pi /3$. Results show that except for closed-loop units with geometrical interference, the normalized area rises from zero (i.e., in-plane fully-folded configuration) up to a turning point (i.e., maximum point), then decreases towards smaller values, and finally follows a plateau regime until the unit reaches the other fully-folded configuration (i.e., out-of-plane). Note that the maximum normalized area occurs at $\beta =\pi /2$, where folding ratio ~ 0.18. This behavior is different for closed-loop units with geometrical interference. For the range of folding ratio at which these units are foldable, the normalized area is greater compared to other units with equal number of sides, *n*, due to a separation between points C and G at all folding ratios (see Fig. 3). Similar behavior is observed for normalized volume [defined as the volume bounded by closed-loop unit (i.e., in-plane cross-sectional area multiplied by the height, *H*), normalized by ${a}^{3}$], except at the folding ratio of 1, at which the normalized volume becomes zero due to the fully-folded configuration of the units, see Fig. 4c.

Next, for folding force calculations, we assume that tubular constructions are made of rigid plates connected together at straight crease lines by linear torsional springs with a spring constant per unit length of $k(N)$. Using the principle of minimum total potential energy on RVE, we derived the following closed-form expression for out-of-plane folding force, $F$ (see Supplementary Information for derivation):

4 $$F=-2kn\,\frac{(\gamma -{\gamma }_{0})\,\frac{a}{b}+(\xi -{\xi }_{0})\,\frac{d\xi }{d\gamma }}{sin(\alpha )\,cos(\gamma /2)},$$

where ${\gamma }_{0}$ and ${\xi }_{0}$ denote the free angles of horizontal and inclined torsional springs, respectively, and $d\xi /d\gamma$ can be calculated using Equation (3). The equation given above clearly show that the folding force is proportional to the number of polygon sides, *n*. For instance, the folding force of an RVE with $n=12$ is three times the force of an RVE with $n=4$ (with same geometrical characteristics).

Figure 4d plots the normalized out-of-plane folding force versus the folding ratio for different values of a/b (ranging from zero to infinity), for all the RVEs studied in this paper. Note that normalization was performed with respect to the spring constant per unit crease length, $k$, and number of polygon sides, $n$, i.e., $F/(k\,n)$. Results were plotted for $\alpha =\pi /3$, and free angle of torsional springs achieved at ${\gamma }_{0}=\pi /3$ (folding ratio = 0.5; ${\beta }_{0}$ and ${\xi }_{0}$ can be calculated from Equation (3) by plugging ${\gamma }_{0}$ instead of $\gamma$). In Fig. 4e, we plotted the normalized out-of-plane folding force versus the folding ratio for a set of $\alpha$, varying between the extreme cases, $\alpha =0$ and $\alpha =\pi /2$ for $\frac{a}{b}=1$ and ${\gamma }_{0}=\pi /3$. Also, to highlight the effect of the free angles of torsional springs, we plot the normalized out-of-plane folding force versus the folding ratio for $\frac{a}{b}=1$, $\alpha =\pi /3$, and different values of ${\gamma }_{0}$ varying between the extreme cases, ${\gamma }_{0}=0$ and ${\gamma }_{0}=\pi$, Fig. 4f. Results clearly show that for ${\gamma }_{0}$ greater than ~ $0.85\,\pi$, a ‘bistability’ (i.e., having two stable configurations) is observed for RVEs under the out-of-plane folding force - independent of the number of polygon sides, $n$. For instance, the RVEs with ${\gamma }_{0}=11\pi /12$, exhibit local extremum points at folding ratios of ~ 0.07 (local maximum) and ~ 0.51 (local minimum). This demonstrates the existence of two stable configurations – one at folding ratio of close to 0, where the folding force is zero, and – the other one at the local minimum point at folding ratio of ~ 0.51. Existence of bistability in certain unit designs highlights their potential application in designing foldable structures for energy absorption, energy harvesting, and impact mitigation^3,12,27,28^.

**Figure 5:**
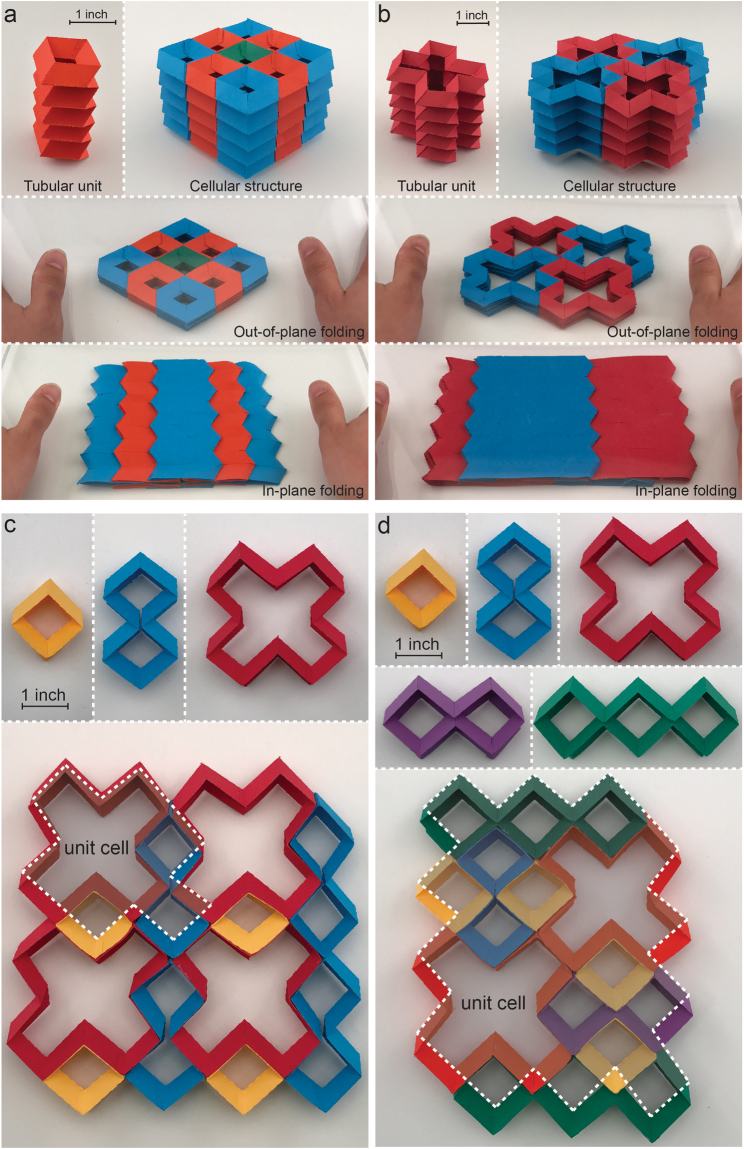
Modular construction of foldable structures using the closed-loop units introduced in this paper as the building blocks. (**a**) and (**b**) Tubular and 3D periodic cellular structures constructed with tessellation of foldable closed-loop units with $n=4$ and $n=12$, respectively. The closed-loop units are first stacked on top of each other – in out-of-plane direction – to create a foldable tubular configuration. Tessellation of these tubular units in 2D in-plane area will form the final assembly of foldable 3D periodic cellular structures. We employed an adhesive material (i.e., glue) to link the adjacent tubular structures together to form the final assemblies. Figures demonstrate the foldability of these 3D cellular structures in both out-of-plane and in-plane folding directions. (**c**) and (**d**) Two examples of modular constructions, each created by employing multiple types of closed-loop units as the building blocks. The closed-loop units for each modular construction are shown on the top of each example.

Tessellation of closed-loop units or tubular constructions in the in-plane direction can create planar and 3D periodic foldable cellular structures, respectively. This results in creation of a wide range of foldable structures with one degree-of-freedom (DoF) with properties that are governed by their building blocks. Figure 5a,b show examples of tubular and 3D periodic cellular structures constructed with tessellation of closed-loop units with $n=4$ (i.e., the smallest foldable closed-loop unit) and $n=12$ (see Supplementary Information for more examples). In each example, the closed-loop units are first stacked on top of each other, in out-of-plane direction, to create a foldable tubular configuration. Tessellation of these tubular units in 2D in-plane area will form the final assembly of foldable 3D periodic cellular structures. Foldability of these 3D cellular structures in out-of-plane and in-plane directions are also demonstrated in both folding directions. Figure 5c,d show two examples of modular constructions made by using more than one type of closed-loop unit. To this end, the ‘unit cells’ are first created by employing multiple types of closed-loop units. Then, a planar tessellation of these unit cells will result in the final assembly of periodic structures. The closed-loop units, as well as the associated unit cell for each modular construction are shown in the figures. Both examples shown are fully-foldable in out-of-plane and in-plane folding directions.

In summary, we presented a set of mathematical expressions to design foldable Miura-based closed-loop origami units, which can further be used as building blocks for modular design of foldable structures including tubular, and 2D and 3D cellular constructions. Almost all the structures given have one DoF with flat-foldability in out-of-plane and in-plane folding directions. The folding kinematics and kinetics of the constructed foldable structures can be tuned through a number of geometrical parameters of their building blocks as well as the specific design (pattern) of the foldable structure. The work presents a novel and application-driven approach for developing origami-based foldable and deployable structures with modular construction through the design of their underlying building blocks.

## Methods

### Fabrication of the origami-based units and structures

All the samples were fabricated out of paper (thickness ~ 0.01 in), where the cuts and crease lines were made using a Silhouette CAMEO cutting machine (Silhouette America, Inc., Lindon, UT). Parts of closed-loop units or tubular constructions that can be made out of a single sheet of paper were first cut, and then folded along predefined crease lines. The folded parts were then glued together to form the final closed-loop or tubular configurations (see Supplementary Information for details). The modular constructions were achieved by gluing the underlying closed-loop or tubular constructions together.

## Electronic supplementary material

Supplementary Information
