# Supplementary material for: Origami-based Building Blocks for Modular Construction of Foldable Structures
Source: Sci Rep. 2017 Nov 1;7:14792. doi: 10.1038/s41598-017-13654-z (PMC5665935; doi:10.1038/s41598-017-13654-z)
Supplement: Supplementary file 1 — Supplementary Information [file 41598_2017_13654_MOESM1_ESM.pdf]

Supplementary Information

Origami-based Building Blocks for Modular Construction of Foldable Structures

Davood Mousanezhad[1], Soroush Kamrava[1], and Ashkan Vaziri[1,*]

[1]Department of Mechanical and Industrial Engineering,
Northeastern University, Boston, MA 02115, USA

[*]Corresponding author email address: vaziri@coe.neu.edu

Contents: Page

1. Number of solutions for Equation (1) 2
2. Derivation of Equation (2) 2
   2.1. Foldability vs. rigidity of closed-loop unit with $n = 4$ 3
3. Details on closed-loop units presented in the manuscript 4
   3.1. Details on internal angles for foldable closed-loop units with n=8 4
   3.2. Details on internal angles for a selected set of foldable closed-loop units with n=12 6
4. Cellular structures made from closed-loop units 8
5. Closed-loop unit fabrication 13
6. Geometrical calculations 14
   6.1. Angle β 14
   6.2. Angle ξ 15
   6.3. Relations between β_1, β_2, and β_3 16
   6.4. Folding ratio 17
7. Force-folding relation 17

1. Number of solutions for Equation (1)

As we mentioned in the manuscript, since the right-hand-side of **Equation (1)** is a constant for an arbitrary closed-loop unit, in order to achieve a foldable configuration, the left-hand-side must be independent of the folding variable, β. This gives us the following equations:

$$(m_1 - m_2 - m_3 + m_4)\,\beta = 0,$$
$$m_2(2\pi) + m_3(\pi) + m_4(\pi) = (m_1 + m_2 + m_3 + m_4 - 2)\,\pi, \quad \text{(S1)}$$

which further give us the following equations:

$$m_1 - m_2 = 2,$$
$$m_3 - m_4 = 2. \quad \text{(S2)}$$

Now, **Equation (S2)** along with the relation, $m_1 + m_2 + m_3 + m_4 = n$, results in:

$$m_2 + m_4 = \frac{n}{2} - 2. \quad \text{(S3)}$$

Since the left-hand-side of this equation is an integer greater than or equal to zero, n must be an even integer greater than or equal to four. Let's substitute $n = 4 + 2p$ (where p is an integer greater than or equal to zero) into **Equation (S3)**. This results in:

$$m_2 + m_4 = p, \quad \text{(S4)}$$

which has $p + 1$ solutions. Now, using the relation between n and p (i.e., $n = 4 + 2p$), the number of solutions for **Equation (1)** becomes $\frac{n}{2} - 1$.

2. Derivation of Equation (2)

As we mentioned in the manuscript, for units given by **Equation (1)** to be closed-loop at an arbitrary folding level (i.e., for any value of the angle, β), the vector summation of the middle crease lines must be equal to zero, i.e., $\sum_{i=1}^{i=n} \overrightarrow{L_\iota} = 0$. Cartesian components of this vector equation can be presented as the following (see **Figure S1**):

$$L_1 - L_2 \cos\theta_{12} + L_3 \cos(\theta_{12} + \theta_{23}) - L_4 \cos(\theta_{12} + \theta_{23} + \theta_{34}) + \cdots = 0,$$
$$L_2 \sin\theta_{12} - L_3 \sin(\theta_{12} + \theta_{23}) + L_4 \sin(\theta_{12} + \theta_{23} + \theta_{34}) - \cdots = 0, \quad \text{(S5)}$$

which can further be reduced to (**Equation (2)** in the main manuscript):

$$\sum_{i=1}^{i=n}(-1)^{i+1} L_i \cos\left(\sum_{j=1}^{j=i-1} \theta_{(j)(j+1)}\right) = 0,$$
$$\sum_{i=2}^{i=n}(-1)^{i} L_i \sin\left(\sum_{j=1}^{j=i-1} \theta_{(j)(j+1)}\right) = 0. \quad \text{(S6)}$$

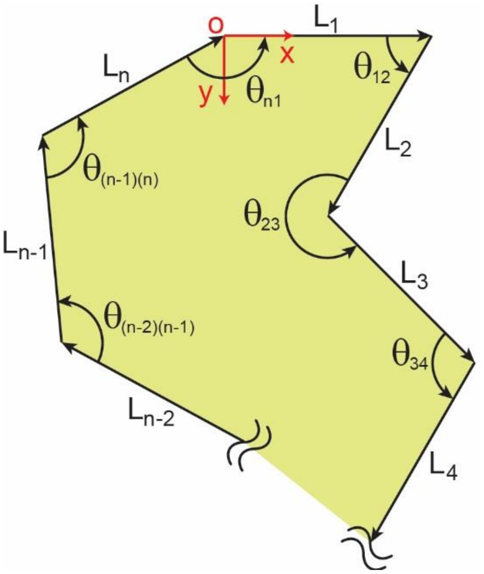

Figure S1. Schematic diagram of the middle crease lines of an arbitrary closed-loop unit (constructed by using fold patterns introduced in **Figure 1**) with n sides, where L_i ($1 \leq i \leq n$ is an integer) is the length of the ith crease line, $\theta_{(j)(j+1)}$ ($1 \leq j \leq n-1$ is an integer) is the internal angle between the jth and $(j+1)^{th}$ crease lines (positive when counterclockwise), and θ_{n1} is the internal angle between the last and first crease lines (positive when counterclockwise).

2.1. Foldability vs. rigidity of closed-loop unit with $n = 4$

Using **Equation (1)**, $n = 4$ results in the smallest foldable closed-loop configuration in the form of 'quadrangle' with two internal angles of β and the other two internal angles of $\pi - \beta$ [i.e., $(m_1, m_2, m_3, m_4) = (2, 0, 2, 0)$]. Here we show that for a quadrangular configuration with angle sequence $(\pi - \beta, \beta, \pi - \beta, \beta)$, applying **Equation (2)** results in $L_1 = L_3$ and $L_2 = L_4$ to satisfy foldability. However, **Equation (2)** cannot be satisfied for a quadrangular configuration with angle sequence $(\pi - \beta, \pi - \beta, \beta, \beta)$ [i.e., rigid unit]. For configuration with sequence $(\pi - \beta, \beta, \pi - \beta, \beta)$, **Equation (2)** gives:

$$L_1 - L_2 \cos(\pi - \beta) + L_3 \cos(\pi) - L_4 \cos(2\pi - \beta) = 0,$$
$$L_2 \sin(\pi - \beta) - L_3 \sin(\pi) + L_4 \sin(2\pi - \beta) = 0, \quad \text{(S7)}$$

which hold true for any value of β if $L_1 = L_3$ and $L_2 = L_4$. On the other hand, for a quadrangular configuration with angle sequence $(\pi - \beta, \pi - \beta, \beta, \beta)$, **Equation (2)** gives:

$$L_1 - L_2 \cos(\pi - \beta) + L_3 \cos(2\pi - 2\beta) - L_4 \cos(2\pi - \beta) = 0,$$
$$L_2 \sin(\pi - \beta) - L_3 \sin(2\pi - 2\beta) + L_4 \sin(2\pi - \beta) = 0, \tag{S8}$$

which do not necessarily hold true for any value of β.

3. Details on closed-loop units presented in the manuscript

3.1. Details on internal angles for foldable closed-loop units with n=8

Tables S1 – S6. Details on internal angles of the closed-loop units shown in **Figure 3**. Alphabets, A-H, label the vertices of the middle crease lines.

Internal angle	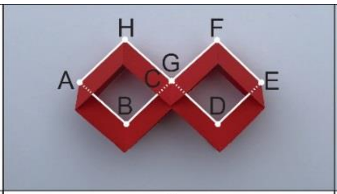	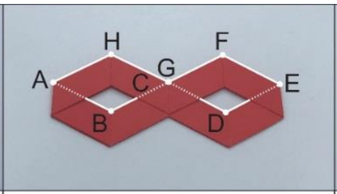	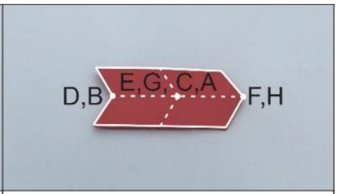
A	β	$\pi - 2\alpha$	π
B	$\pi - \beta$	2α	0
C	$\pi + \beta$	$2\pi - 2\alpha$	2π
D	$\pi - \beta$	2α	0
E	β	$\pi - 2\alpha$	π
F	$\pi - \beta$	2α	0
G	$\pi + \beta$	$2\pi - 2\alpha$	2π
H	$\pi - \beta$	2α	0

Internal angle	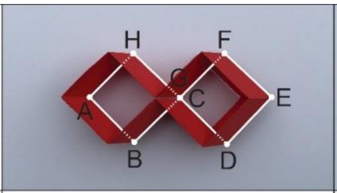	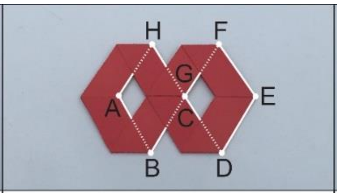	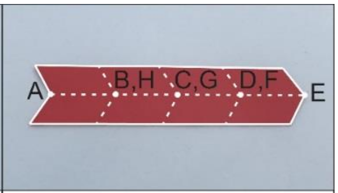
A	$\pi - \beta$	2α	0
B	β	$\pi - 2\alpha$	π
C	$2\pi - \beta$	$\pi + 2\alpha$	π
D	β	$\pi - 2\alpha$	π
E	$\pi - \beta$	2α	0
F	β	$\pi - 2\alpha$	π
G	$2\pi - \beta$	$\pi + 2\alpha$	π
H	β	$\pi - 2\alpha$	π

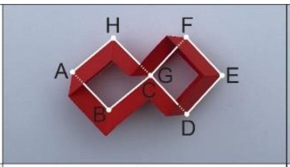

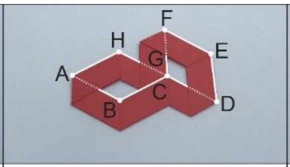

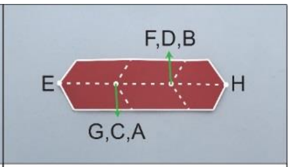

Internal angle			
A	β	$\pi - 2\alpha$	π
B	$\pi - \beta$	2α	0
C	$\pi + \beta$	$2\pi - 2\alpha$	2π
D	β	$\pi - 2\alpha$	π
E	$\pi - \beta$	2α	0
F	β	$\pi - 2\alpha$	π
G	$2\pi - \beta$	$\pi + 2\alpha$	π
H	$\pi - \beta$	2α	0

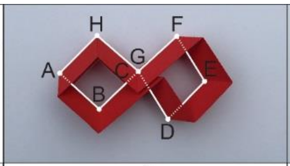

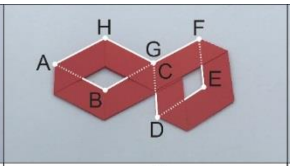

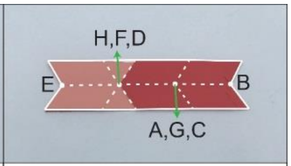

Internal angle			
A	β	$\pi - 2\alpha$	π
B	$\pi - \beta$	2α	0
C	$2\pi - \beta$	$\pi + 2\alpha$	π
D	β	$\pi - 2\alpha$	π
E	$\pi - \beta$	2α	0
F	β	$\pi - 2\alpha$	π
G	$\pi + \beta$	$2\pi - 2\alpha$	2π
H	$\pi - \beta$	2α	0

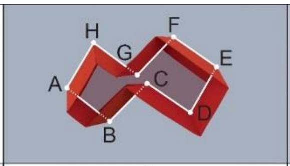

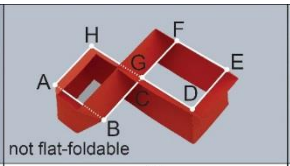

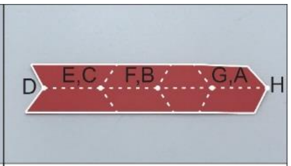

Internal angle			
A	β	$\pi/2$	π
B	β	$\pi/2$	π
C	$2\pi - \beta$	$3\pi/2$	π
D	$\pi - \beta$	$\pi/2$	0
E	β	$\pi/2$	π
F	β	$\pi/2$	π
G	$2\pi - \beta$	$3\pi/2$	π
H	$\pi - \beta$	$\pi/2$	0

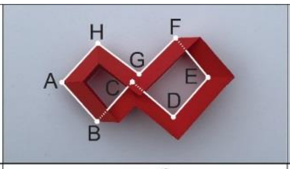

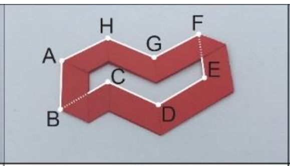

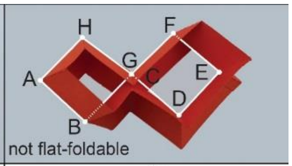

Internal angle			
A	$\pi - \beta$	2α	$\pi/2$
B	β	$\pi - 2\alpha$	$\pi/2$
C	$\pi + \beta$	$2\pi - 2\alpha$	$3\pi/2$
D	$\pi - \beta$	2α	$\pi/2$
E	$\pi - \beta$	2α	$\pi/2$
F	β	$\pi - 2\alpha$	$\pi/2$
G	$\pi + \beta$	$2\pi - 2\alpha$	$3\pi/2$
H	$\pi - \beta$	2α	$\pi/2$

3.2. Details on internal angles for a selected set of foldable closed-loop units with n=12

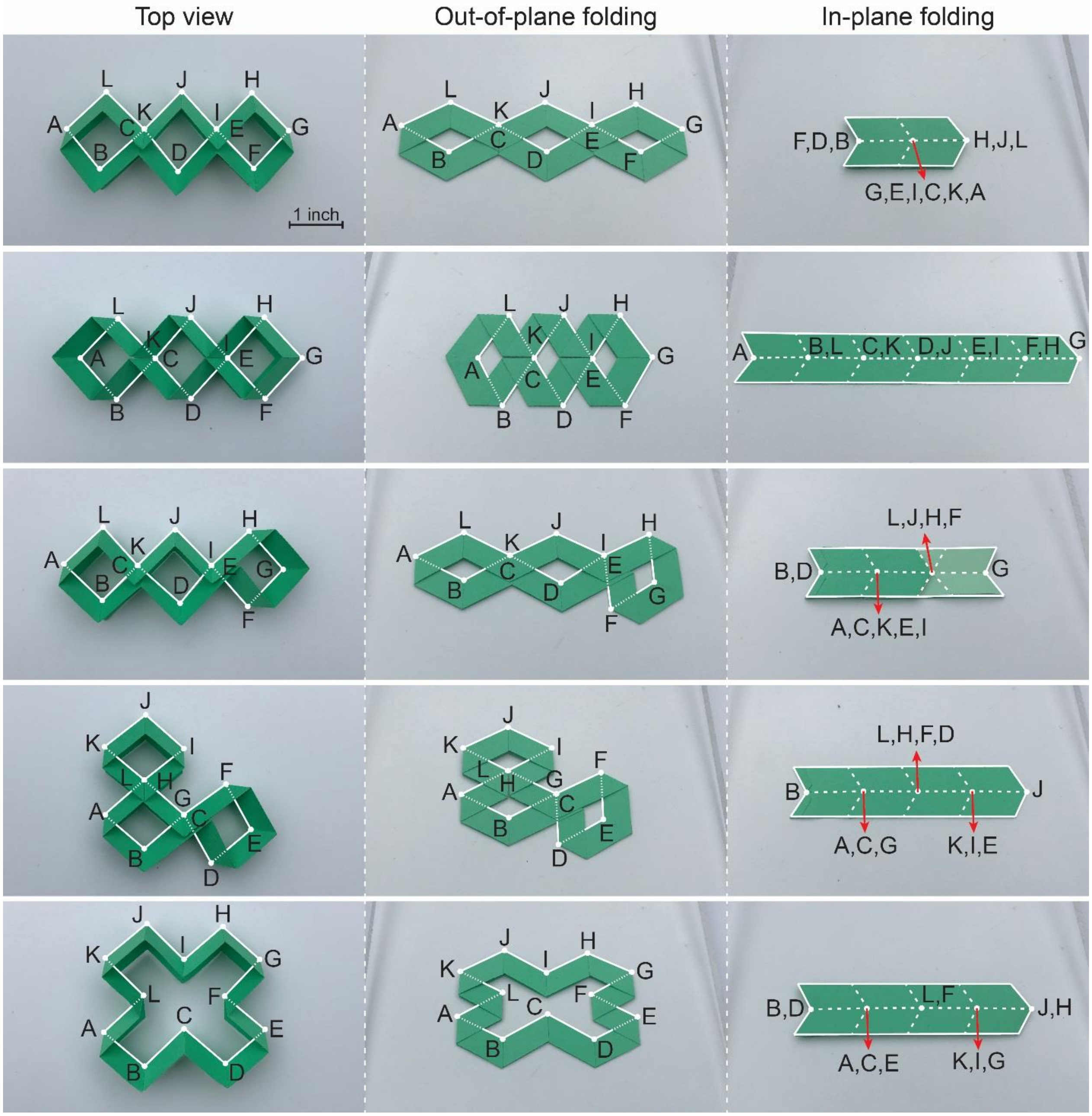

Figure S2. A selected set of foldable closed-loop units with $n = 12$, constructed by using fold patterns introduced in Figure 1.

All the units are one DoF foldable in out-of-plane and in-plane directions, with $L_1 = L_2 = \cdots = L_{12}$ ($= 1$ inch for the samples shown). The sequence of internal angles for each unit is given in Supplementary Information. For the samples shown, $\alpha = \pi/3$. Left column shows a top view of each unit at an unfolded configuration, while middle and right columns show top and side views of units at fully-folded configurations under out-of-plane ($\beta = \pi - 2\alpha$) and in-plane ($\beta = \pi$) directions. We used alphabets, A-L, to label the vertices of the middle crease lines.

Tables S7 – S11. Details on internal angles of the closed-loop units shown in **Figure S2**. Alphabets, A-L, label the vertices of the middle crease lines.

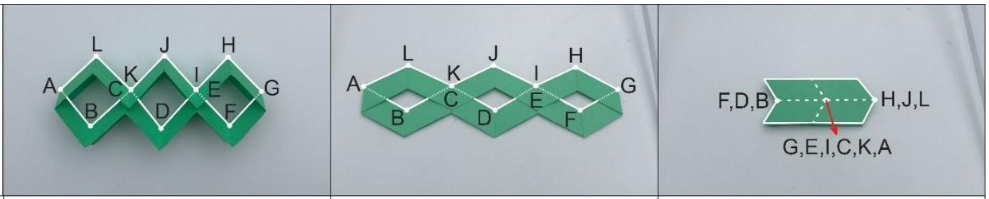

Internal angle			
A	β	$\pi - 2\alpha$	π
B	$\pi - \beta$	2α	0
C	$\pi + \beta$	$2\pi - 2\alpha$	2π
D	$\pi - \beta$	2α	0
E	$\pi + \beta$	$2\pi - 2\alpha$	2π
F	$\pi - \beta$	2α	0
G	$\pi - \beta$	$\pi - 2\alpha$	π
H	$\pi - \beta$	2α	0
I	$\pi + \beta$	$2\pi - 2\alpha$	2π
J	$\pi - \beta$	2α	0
K	$\pi + \beta$	$2\pi - 2\alpha$	2π
L	$\pi - \beta$	2α	0

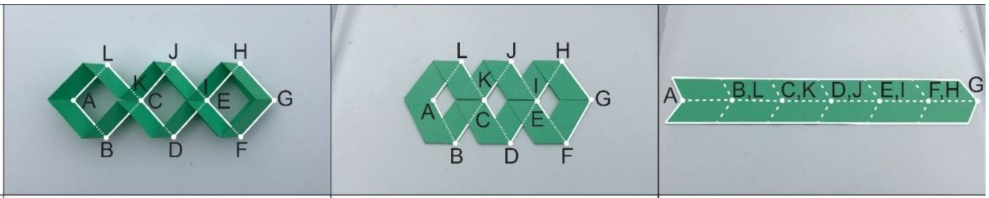

Internal angle			
A	$\pi - \beta$	2α	0
B	β	$\pi - 2\alpha$	π
C	$2\pi - \beta$	$\pi + 2\alpha$	π
D	β	$\pi - 2\alpha$	π
E	$2\pi - \beta$	$\pi + 2\alpha$	π
F	β	$\pi - 2\alpha$	π
G	$\pi - \beta$	2α	0
H	β	$\pi - 2\alpha$	π
I	$2\pi - \beta$	$\pi + 2\alpha$	π
J	β	$\pi - 2\alpha$	π
K	$2\pi - \beta$	$\pi + 2\alpha$	π
L	β	$\pi - 2\alpha$	π

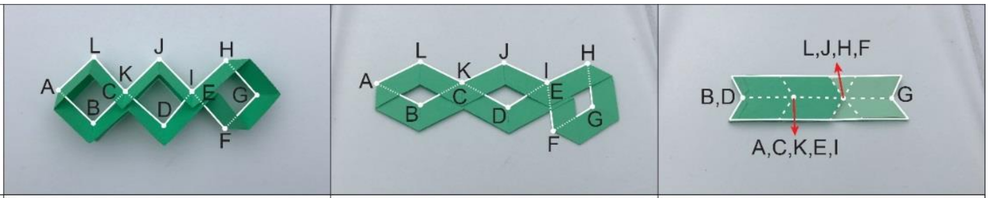

Internal angle			
A	β	$\pi - 2\alpha$	π
B	$\pi - \beta$	2α	0
C	$\pi + \beta$	$2\pi - 2\alpha$	2π
D	$\pi - \beta$	2α	0
E	$2\pi - \beta$	$\pi + 2\alpha$	π
F	β	$\pi - 2\alpha$	π
G	$\pi - \beta$	2α	0
H	β	$\pi - 2\alpha$	π
I	$\pi + \beta$	$2\pi - 2\alpha$	2π
J	$\pi - \beta$	2α	0
K	$\pi + \beta$	$2\pi - 2\alpha$	2π
L	$\pi - \beta$	2α	0

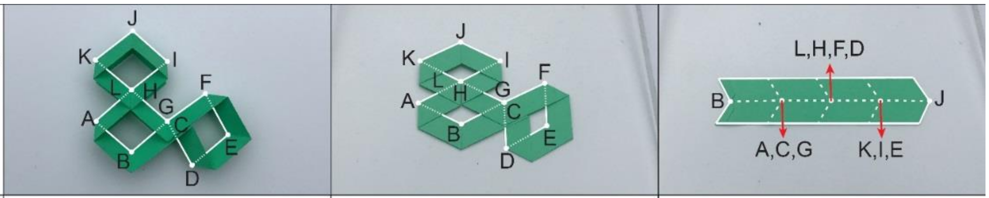

Internal angle			
A	β	$\pi - 2\alpha$	π
B	$\pi - \beta$	2α	0
C	$2\pi - \beta$	$\pi + 2\alpha$	π
D	β	$\pi - 2\alpha$	π
E	$\pi - \beta$	2α	0
F	β	$\pi - 2\alpha$	π
G	$\pi + \beta$	$2\pi - 2\alpha$	2π
H	$2\pi - \beta$	$\pi + 2\alpha$	π
I	β	$\pi - 2\alpha$	π
J	$\pi - \beta$	2α	0
K	β	$\pi - 2\alpha$	π
L	$2\pi - \beta$	$\pi + 2\alpha$	π

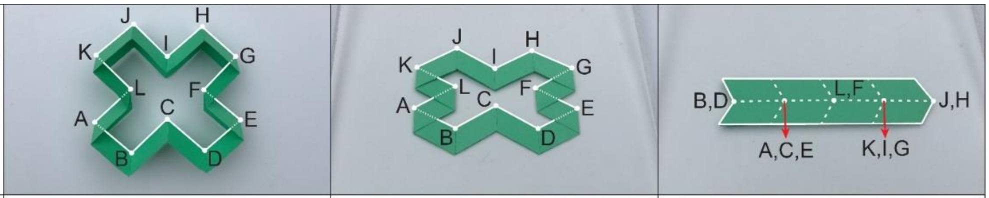

Internal angle			
A	β	$\pi - 2\alpha$	π
B	$\pi - \beta$	2α	0
C	$\pi + \beta$	$2\pi - 2\alpha$	2π
D	$\pi - \beta$	2α	0
E	β	$\pi - 2\alpha$	π
F	$2\pi - \beta$	$\pi + 2\alpha$	π
G	β	$\pi - 2\alpha$	π
H	$\pi - \beta$	2α	0
I	$\pi + \beta$	$2\pi - 2\alpha$	2π
J	$\pi - \beta$	2α	0
K	β	$\pi - 2\alpha$	π
L	$2\pi - \beta$	$\pi + 2\alpha$	π

4. Cellular structures made from closed-loop units

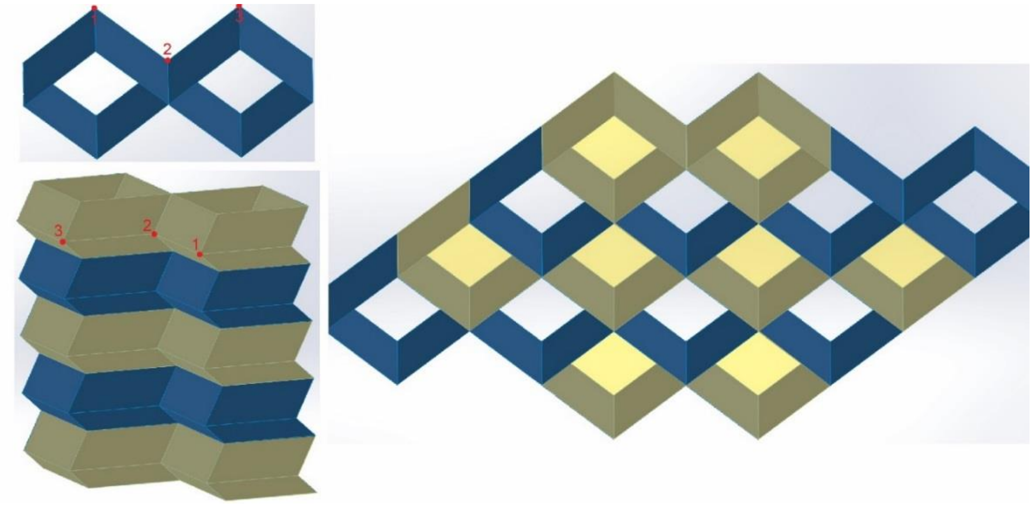

Figure S3. 3D CAD models of closed-loop unit, tubular construction, and 3D periodic cellular structure constructed with tessellation of foldable closed-loop unit shown in **Figure 3** – first row.

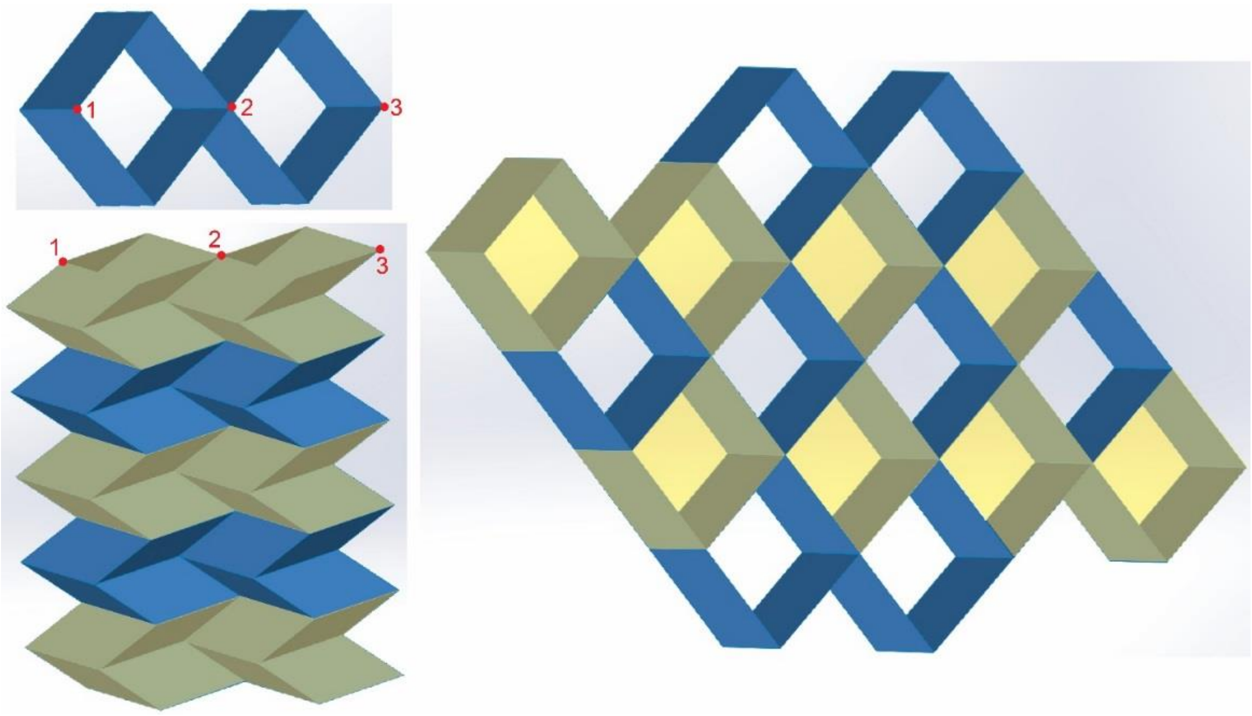

Figure S4. 3D CAD models of closed-loop unit, tubular construction, and 3D periodic cellular structure constructed with tessellation of foldable closed-loop unit shown in **Figure 3** – second row.

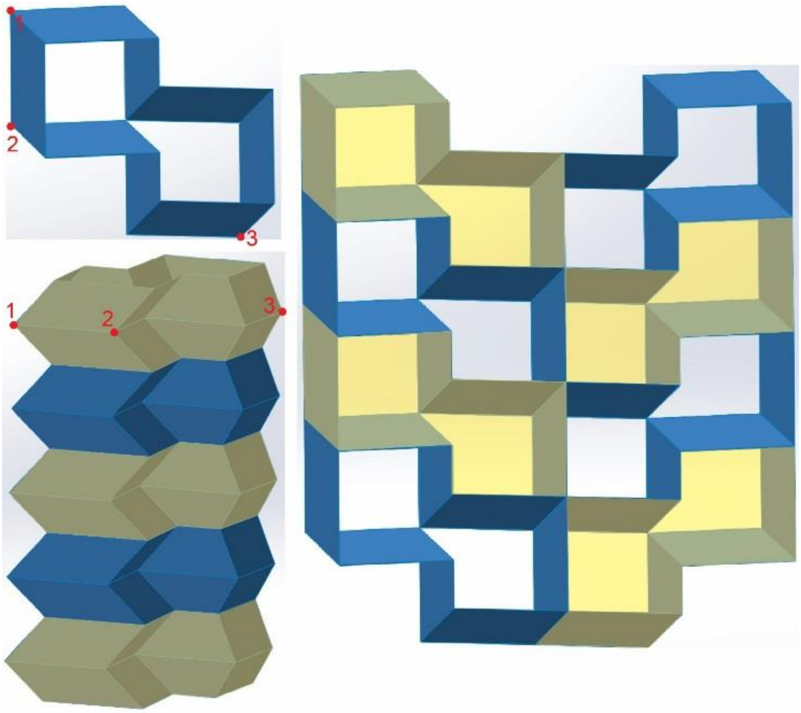

Figure S5. 3D CAD models of closed-loop unit, tubular construction, and 3D periodic cellular structure constructed with tessellation of foldable closed-loop unit shown in **Figure 3** – third row.

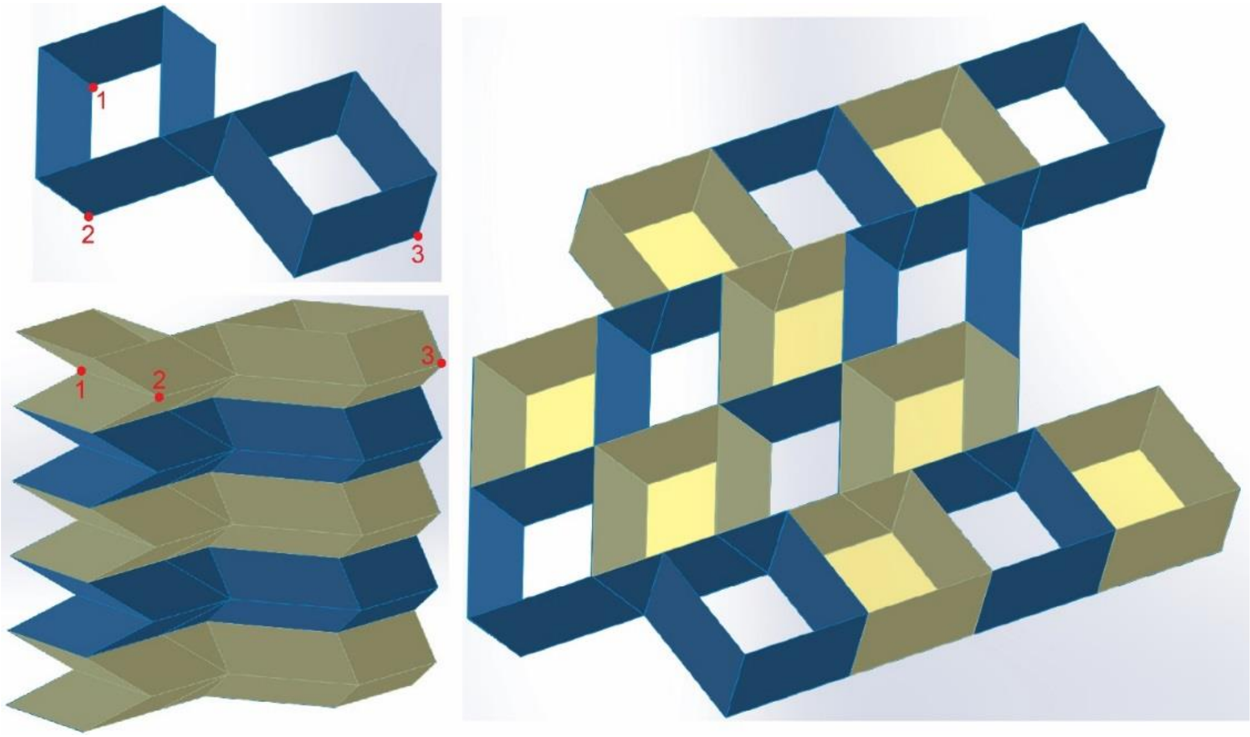

Figure S6. 3D CAD models of closed-loop unit, tubular construction, and 3D periodic cellular structure constructed with tessellation of foldable closed-loop unit shown in **Figure 3** – fourth row.

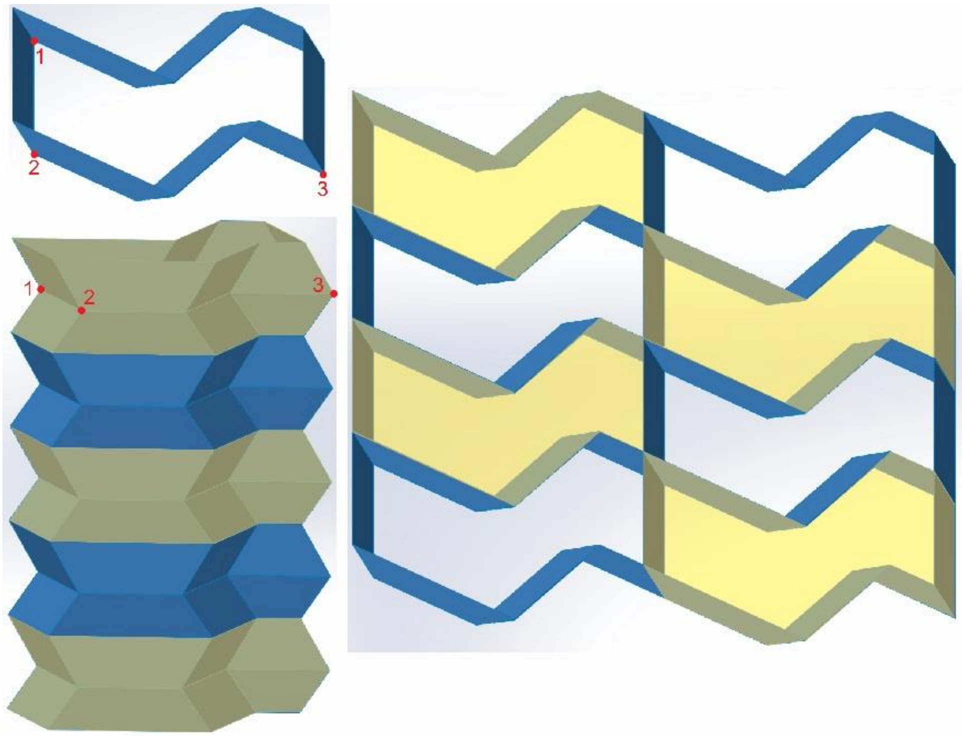

Figure S7. 3D CAD models of closed-loop unit, tubular construction, and 3D periodic cellular structure constructed with tessellation of foldable closed-loop unit shown in **Figure 3** – fifth row.

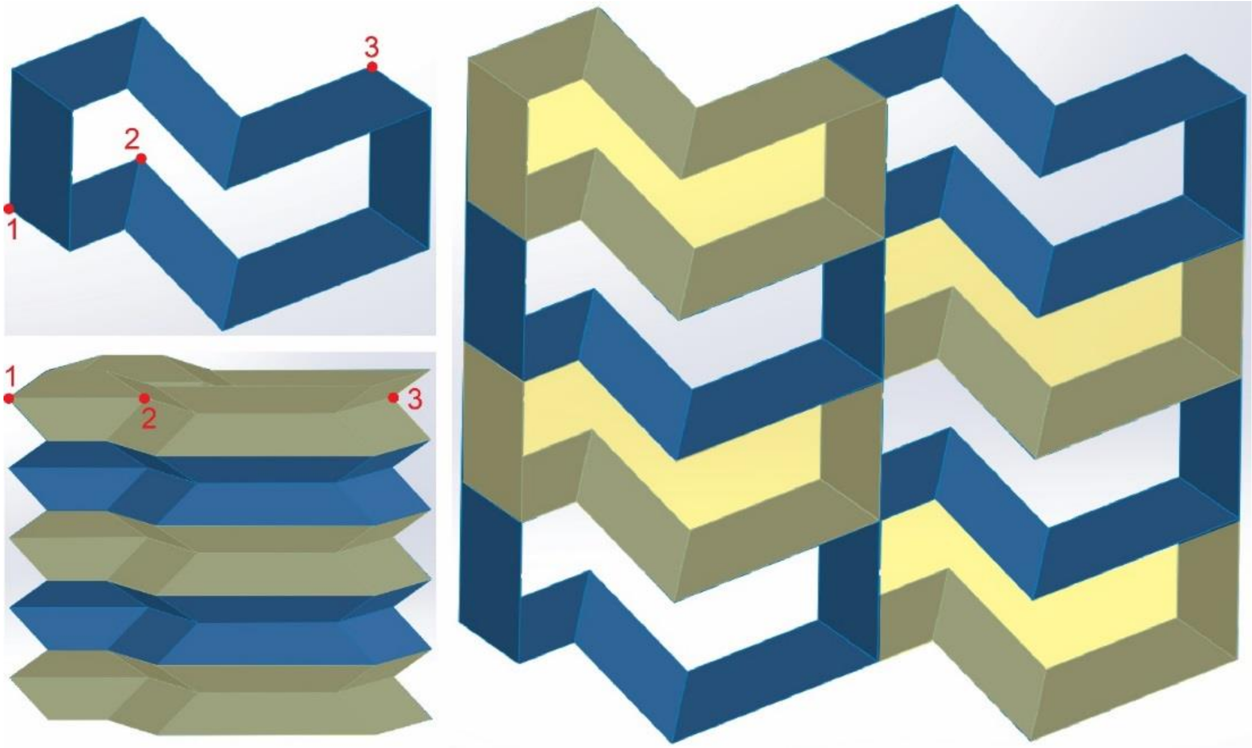

Figure S8. 3D CAD models of closed-loop unit, tubular construction, and 3D periodic cellular structure constructed with tessellation of foldable closed-loop unit shown in **Figure 3** – sixth row.

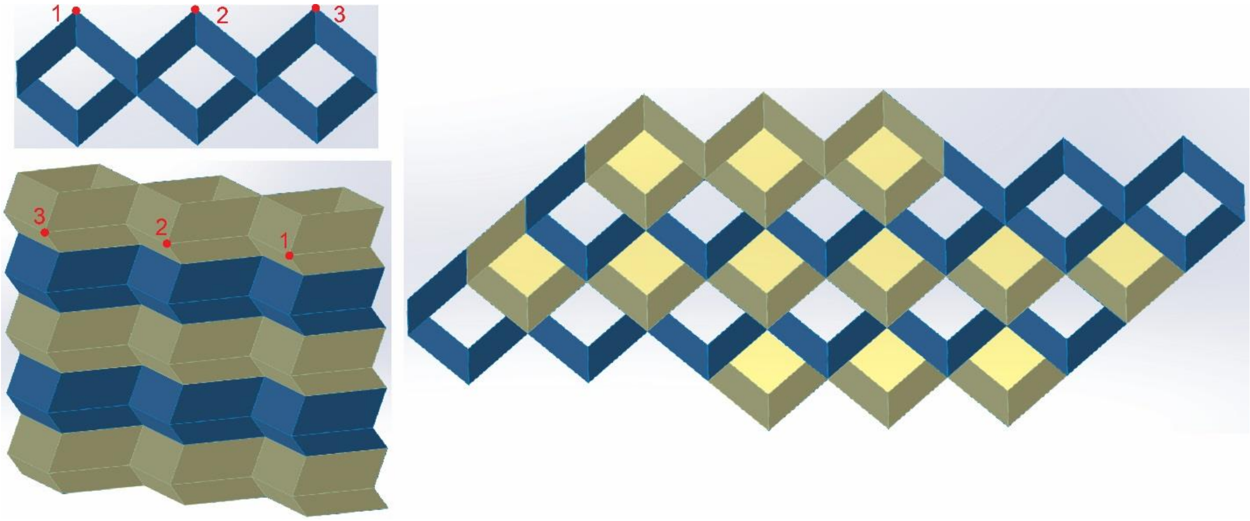

Figure S9. 3D CAD models of closed-loop unit, tubular construction, and 3D periodic cellular structure constructed with tessellation of foldable closed-loop unit shown in **Figure 4** – first row.

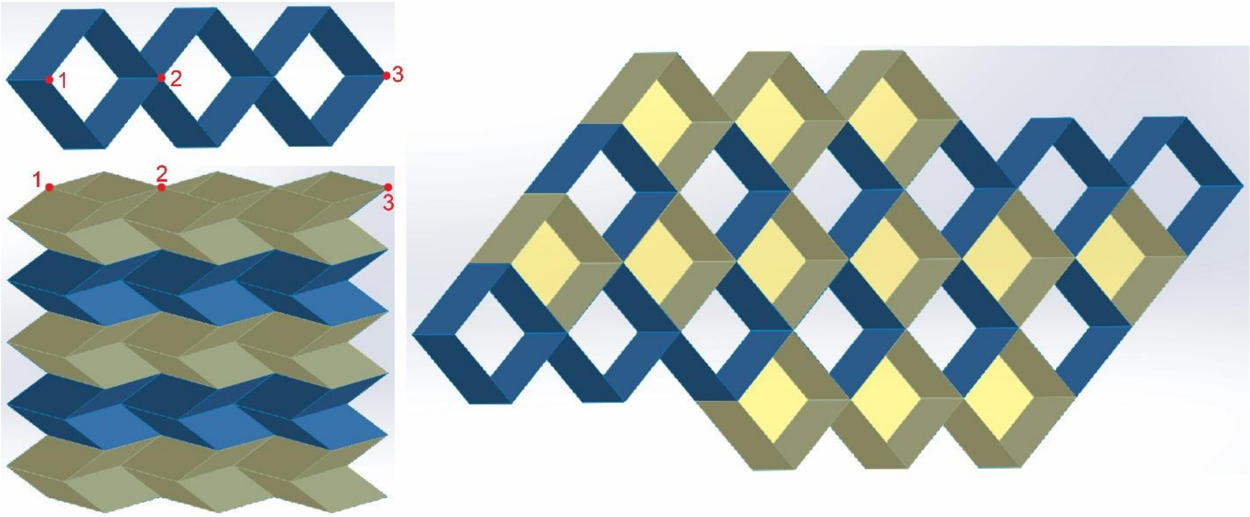

Figure S10. 3D CAD models of closed-loop unit, tubular construction, and 3D periodic cellular structure constructed with tessellation of foldable closed-loop unit shown in **Figure 4** – second row.

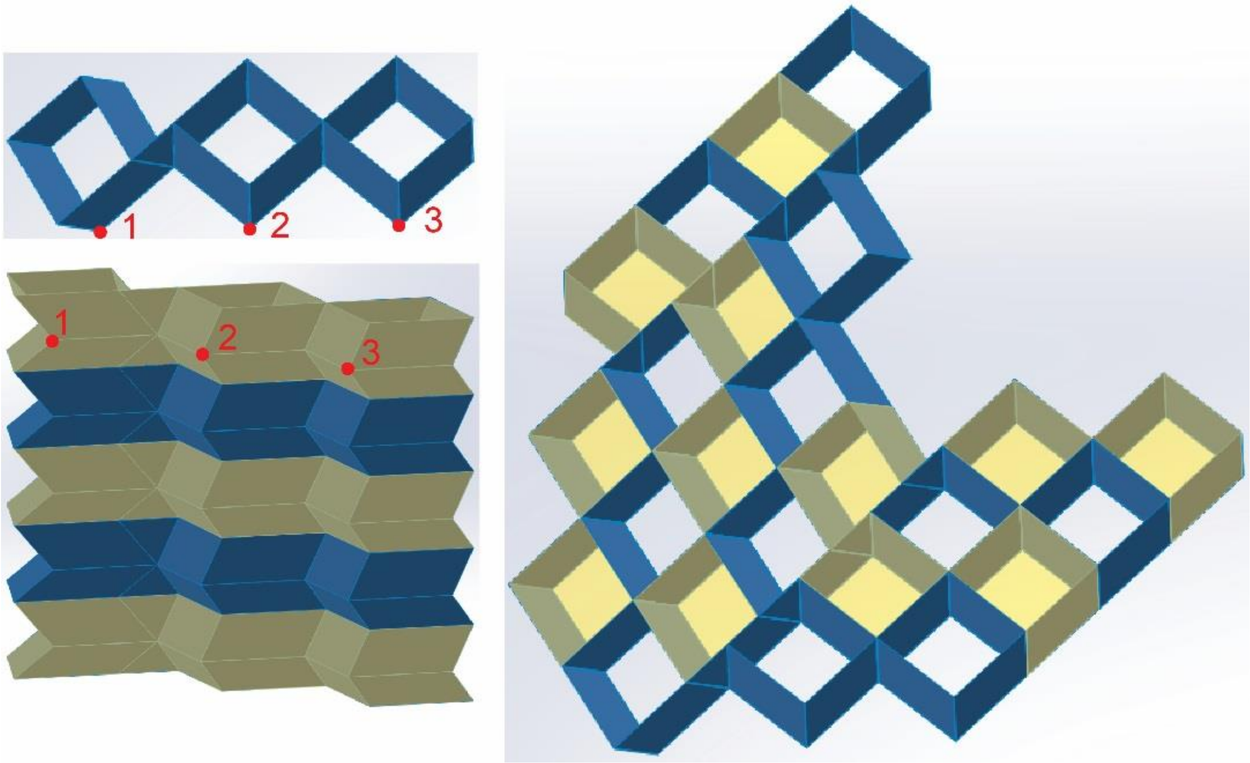

Figure S11. 3D CAD models of closed-loop unit, tubular construction, and 3D periodic cellular structure constructed with tessellation of foldable closed-loop unit shown in **Figure 4** – third row.

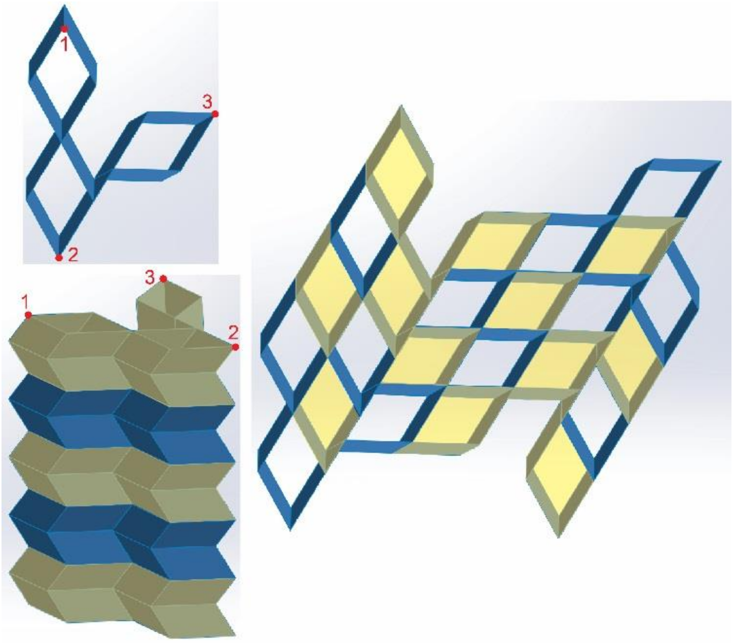

Figure S12. 3D CAD models of closed-loop unit, tubular construction, and 3D periodic cellular structure constructed with tessellation of foldable closed-loop unit shown in **Figure 4** – fourth row.

5. Fabrication of closed-loop units

Figure S13 shows the steps of construction of a quadrangular closed-loop unit as: cut, fold, and glue. Parts of the unit that can be made out of a single sheet of paper are first cut, and then folded along predefined crease lines. The folded parts are then glued together to form the final closed-loop configuration.

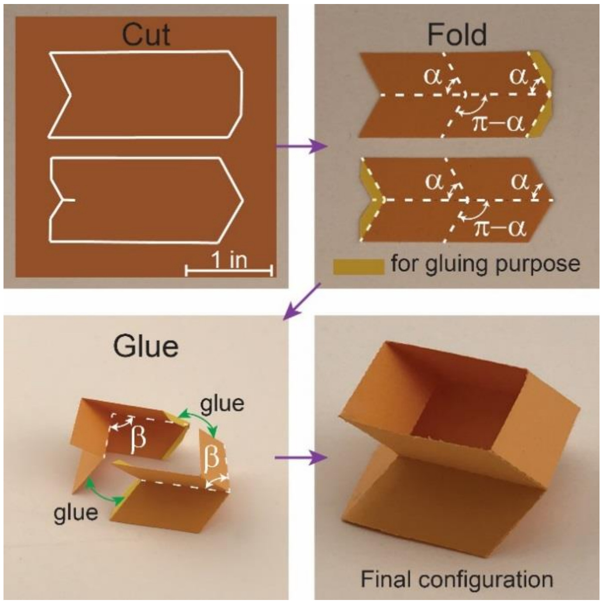

Figure S13. Steps of construction of closed-loop units out of multiple sheets of paper.

6. Geometrical calculations

6.1. Angle $\boldsymbol{\beta}$

The configuration of a Miura-ori unit (i.e., half of quadrangular closed-loop unit shown in **Figure 2b**) at an arbitrary folding level can be fully quantified by an angular value defining the coordinate of the single degree-of-freedom (DoF) of the unit. This angular value can be chosen between the two dihedral angles, $\gamma \in [0, \pi]$, and $\xi \in [0, \pi]$, or the angle between the mountain and valley folds, $\beta \in [\pi - 2\alpha, \pi]$, see **Figure S14a**. Now, considering γ as an independent parameter, we can obtain β and ξ as functions of γ (i.e., β and ξ will be considered as dependent parameters). We now begin the analysis by calculating the vectors $\overrightarrow{AB}$ and $\overrightarrow{AC}$ as the following (see **Figure S14a**):

$$\begin{aligned} \overrightarrow{AB} &= +b\cos\phi\,\vec{\imath} + b\sin\phi\,\vec{\jmath}, \\ \overrightarrow{AC} &= -a\cos\psi\,\vec{\imath} - a\sin\psi\,\vec{k}, \end{aligned} \quad \text{(S9)}$$

where a and b are side lengths, and $\vec{\imath}$, $\vec{\jmath}$, and $\vec{k}$ are unit vectors along the *x*, *y*, and *z* directions, respectively. Now, the following expression defines the angle between the vectors $\overrightarrow{AB}$ and $\overrightarrow{AC}$:

$$\cos^{-1}\left(\frac{\overrightarrow{AB}.\overrightarrow{AC}}{|\overrightarrow{AB}||\overrightarrow{AC}|}\right) = \pi - \alpha. \quad \text{(S10)}$$

Next, substituting **Equation (S9)** into **Equation (S10)** will result in the following relation between angles ϕ, ψ, and α:

$$\cos\phi\ \cos\psi = \cos\alpha. \quad \text{(S11)}$$

Now, considering the isosceles triangles, *ABF* and *AED* (see **Figure S14b**), the following relations can be obtained for the angles γ and ϕ:

$$\begin{aligned} \sin(\gamma/2) &= \frac{\overline{DE}/2}{\overline{AE}}, \\ \sin\phi &= \frac{\overline{BF}/2}{\overline{AB}}, \end{aligned} \quad \text{(S12)}$$

where $\overline{DE}$ is the length of the edge DE (similarly for other edges). We should note that $\overline{AE} = \overline{AB}\sin\alpha$, and $\overline{BF} = \overline{DE}$, which by substituting into **Equation (S12)** will result in the following:

$$\sin\alpha\ \sin(\gamma/2) = \sin\phi. \tag{S13}$$

Finally, **Figure S14a** shows that $\beta = \pi - 2\psi$, which by using **Equations (S11)** and **(S13)** will result in the following equation for β:

$$\beta = \pi - 2\cos^{-1}\left(\frac{\cos\alpha}{\sqrt{1-\sin^2\alpha\,\sin^2(\gamma/2)}}\right). \tag{S14}$$

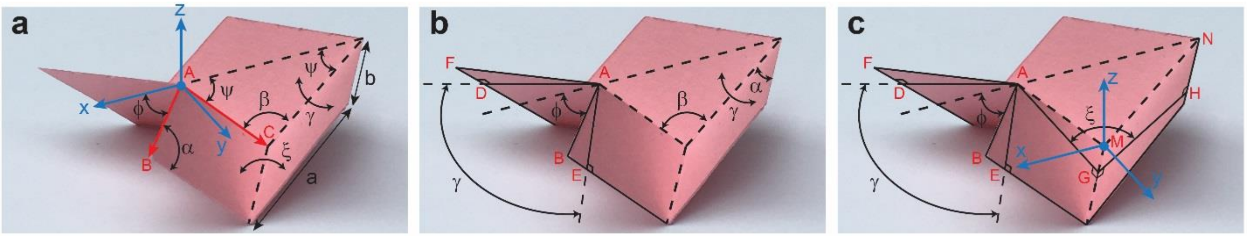

Figure S14. Geometrical characteristics of a Miura-ori fold pattern at an arbitrary fold pattern.

6.2. Angle ξ

In order to obtain a closed-form expression for the angle, ξ, as a function of γ, we first translate (with no rotations) the coordinate system of **Figure 14a** from point *A* to point *M*, see **Figure 14c**. Note that the angle ξ is basically the angle between vectors $\overrightarrow{GA}$ and $\overrightarrow{GH}$. We now begin the analysis by obtaining the coordinates of points *G*, *A*, and *H* with respect to the new coordinate system located at point *M*, as the following:

$$G = \begin{bmatrix} X_G \\ Y_G \\ Z_G \end{bmatrix} = \begin{bmatrix} a\cos\alpha\cos\phi \\ a\cos\alpha\sin\phi \\ 0 \end{bmatrix},$$
$$A = \begin{bmatrix} X_A \\ Y_A \\ Z_A \end{bmatrix} = \begin{bmatrix} a\sin(\beta/2) \\ 0 \\ a\cos(\beta/2) \end{bmatrix},$$
$$H = \begin{bmatrix} X_H \\ Y_H \\ Z_H \end{bmatrix} = \begin{bmatrix} -a\sin(\beta/2) + 2a\cos\alpha\cos\phi \\ 2a\cos\alpha\sin\phi \\ a\cos(\beta/2) \end{bmatrix}. \tag{S15}$$

Note that we employed the relation, $\overline{NH} = 2\overline{MG} = 2a\cos\alpha$, to derive the set of coordinates presented in **Equation (S15)**. Next, using **Equation (S15)** the vectors, $\overrightarrow{GA}$ and $\overrightarrow{GH}$, will be determined as the following:

$$\overrightarrow{GA} = \begin{bmatrix} a\sin(\beta/2) - a\cos\alpha\cos\phi \\ -a\cos\alpha\sin\phi \\ a\cos(\beta/2) \end{bmatrix},$$
$$\overrightarrow{GH} = \begin{bmatrix} -a\sin(\beta/2) + a\cos\alpha\cos\phi \\ a\cos\alpha\sin\phi \\ a\cos(\beta/2) \end{bmatrix}. \tag{S16}$$

Next, we calculate the angle ξ as:

$$\cos\xi = \left(\frac{\overrightarrow{GA}.\overrightarrow{GH}}{|\overrightarrow{GA}||\overrightarrow{GH}|}\right) = \frac{\cos\beta - \cos^2\alpha + 2\cos\alpha\cos\phi\sin(\beta/2)}{1+\cos^2\alpha - 2\cos\alpha\cos\phi\sin(\beta/2)}, \tag{S17}$$

which can further be simplified [by using **Equations (S13)** and **(S14)**] into the following:

$$\xi = \cos^{-1}\left(\frac{1-(1+\cos^2\alpha)\ \sin^2(\gamma/2)}{1-\sin^2\alpha\ \sin^2(\gamma/2)}\right). \tag{S18}$$

6.3. Relations between $\boldsymbol{\beta_1}$, $\boldsymbol{\beta_2}$, and $\boldsymbol{\beta_3}$

Figure S15a shows a schematic diagram of a unit constructed by crease patterns shown in **Figures 1a** and **1c**. Note that the triangular faces, 1, 2, and 3 – highlighted by grey – are always parallel to each other. This leads to $\delta_1 = \delta_2$ and $\delta_3 = \eta_2$. Furthermore, due to rotational symmetries, $\delta_1 = \delta_3$, and due to mirror symmetries, $\eta_1 = \eta_2$. So, we conclude that $\delta_1 = \delta_2 = \delta_3 = \eta_1 = \eta_2$. Note that $\eta_1 + \eta_2 = \beta_2$ and $\delta_2 + \delta_3 = \pi - \beta_1$, which result in $\beta_2 = \pi - \beta_1$. Similarly, we can show that $\beta_3 = \pi - \beta_1$ (see **Figure S15b**).

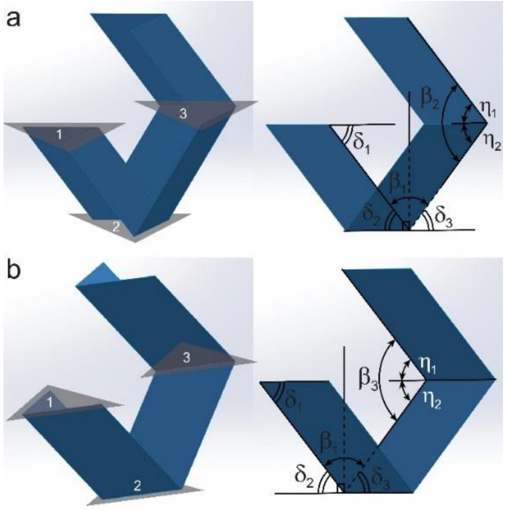

Figure S15. Schematic diagram of units constructed by crease patterns shown in **Figure 1**.

6.4. Folding ratio

Unit height, H, is equal to $\overline{BF}$ $(= \overline{DE})$ [see **Figure S14**] and can be given as the following:

$$H = 2b\sin(\alpha)\sin(\gamma/2). \tag{S19}$$

We define folding ratio as $-[H - H_0]/H_0$ (similar to uniaxial out-of-plane compressive strain), where H_0 is the unit height at $\gamma = \pi$ (i.e., in-plane fully-folded configuration). Using **Equation (S19)**, folding ratio can be obtained as $1 - \sin(\gamma/2)$.

7. Force-folding relation

Here, we calculate the out-of-plane folding force needed to achieve a desired folding level. As we mentioned in the manuscript, the constructions are assumed to be made of rigid plates connected together at straight crease lines by linear torsional springs with a spring constant per unit length of $k(N)$. The RVE in our analysis is a single closed-loop unit with crease lines at top and bottom (i.e., at the lines joining to the upper and lower units to form a tubular construction) modelled as torsional springs with a spring constant per unit length of $k/2$ (N). Now, we calculate the total potential energy stored in torsional springs, U, and the external work done by external force, W, while the DoF of the RVE goes from γ_0 (i.e., initial folding angle of torsional springs) to γ (i.e., desired folding level):

$$U = 2(n_1 + n_2 + n_3)\times\frac{1}{2}k(\gamma-\gamma_0)^2\times a + 2n_1\times\frac{1}{2}k(\xi-\xi_0)^2\times b +$$
$$2(n_2+n_3)\times\frac{1}{2}k(\pi-\xi-(\pi-\xi_0))^2\times b,$$
$$W = F\times\Delta H = F\times\big(2b\,sin\,\alpha\,sin(\gamma_0/2) - 2b\,sin\,\alpha\;\;sin(\gamma/2)\big), \tag{S20}$$

where n_1, n_2, and n_3 are the number of fold patterns shown in **Figures 1a**, **1b**, and **1c**, respectively. Using **Equation (S20)** and the principle of minimum total potential energy ($\partial\pi/\partial\gamma = 0$, where $\pi = U - W$), F can be calculated as:

$$F = -\,2kn\,\frac{(\gamma-\gamma_0)\frac{a}{b} + (\xi-\xi_0)\frac{d\xi}{d\gamma}}{\sin(\alpha)\,\cos(\gamma/2)}, \tag{S21}$$

where $n = n_1 + n_2 + n_3$.
